# An innovative full-size pathogenic tandem duplication mutation precise detection system based on next-generation sequencing

**DOI:** 10.3389/ebm.2025.10128

**Published:** 2025-07-11

**Authors:** Li-Li Zhang, Zhe Wang, Ying Zhou, Dai-Yang Li, Xiao-Nian Tu, Yu-Xia Li, Ke-Ming Du, Zhong-Zheng Zheng

**Affiliations:** Shanghai Tissuebank Biotechnology Co., Ltd., Shanghai, China

**Keywords:** internal tandem duplication, next-generation sequencing, acute myeloid leukemia, prognosis, mutation detection

## Abstract

Accurate identifying internal tandem duplication (ITD) mutation is indispensable for diagnosis and prognosis of acute myeloid leukemia (AML) patients, but specialized full-size detection tools are lacking. Therefore, we aimed to develop a reliable system for accurate assessment of ITD mutations of various size ranges and improve prognosis for AML. Bone marrow samples from AML patients from December 2021 to March 2022 were collected for methodology establishment. After a large-scale sample testing by next-generation sequencing (NGS), a short-read tandem duplication recognition system based on soft-clip was established. During performance validation, the lower detection limit was set to a parameter close to capillary electrophoresis (“gold standard”) by adjusting reference values (sensitivity 3–5%). Data simulation was performed using the FLT3 gene CDS as wild-type data. Methodological concordance of this system with capillary electrophoresis was analyzed. The applicability to other pathogenic tandem duplication mutations was validated. We have developed an innovative NGS-based system named “ITDFinder” for accurate detection of ITD mutations, with the lower detection limit of 4%, corresponding to a sequencing depth of 1000X. Compared to capillary electrophoresis, ITDFinder exhibited good consistency (mean difference: −0.0085) in mutation detection and correlation across various length of ITD. Clinical case validation (n = 1,032) showed an overall agreement rate of 96.5% between the two approaches used for characterization. In addition, data simulation results suggested that the new system could observe BCOR-ITD and KMT2A-PTD mutations (depths, 500–1300X; mutation rates, 0.04–0.8). The innovative mutation detection system is appropriate to small-to large-sized ITDs and other pathogenic tandem duplication mutations, expected to save 96.3% of the workload. This offers significant potential for accurate clinical assessment of ITD mutations and subsequent prognosis in AML patients.

## Impact statement

We developed a new NGS-based pathogenic tandem repeat mutation precision detection system named “ITDFinder” that goes beyond capillary electrophoresis and towards multi-sized mutation length. NGS enables identification of single nucleotide mutations and gene mutations with lower detection limits and provides more objective quantification of FLT3-ITD allele load, with the advantages of short run time, low testing cost for large-scale samples, and flexible library preparation and analysis strategies to address the challenges of challenging genomic fractions. The ITDFinder system has two key advantages: first, the negative results from this ITDFinder system can be approximately equivalent to negative capillary electrophoresis results, eliminating the need for capillary electrophoresis experiments (nearly 97% of these samples based on high-volume sample validation) and shortening the NGS experimental cycle. The ITDFinder system can also accurately detect tandem repeat mutation ratios in other disorders, such as BCOR-ITD and KMT2A-PTD.

## Introduction

Acute myeloid leukemia (AML) is a common malignant hematologic neoplasm caused by complementary functional pathogenic gene mutations leading to uncontrolled proliferation and maturation arrest of bone marrow progenitor cells. The FMS-like tyrosine kinase 3 (FLT3)-internal tandem duplication (ITD) is one of the most frequent mutations in AML (up to 25–30% mutation rate [1, 2]). Selection of resistant FLT3 clones, avoidance of FLT3 inhibition, or insufficient therapeutic response all contribute to FLT3-ITD persistence [3, 4]. Genetic aberrations permit accurate categorization with hazard evaluation in 50–55% of AML cases [5]. Therefore, accurate identification and mutation assessment of AML-causing genes such as ITD is indispensable for the diagnosis, treatment and prognosis of patients.

The “gold standard” for quantifying ITD mutations in clinical practice has been viewed as capillary electrophoresis [6], but it solely provides data on mutation frequency and sequence length, not other ITD-related information like the precise sequence of the insertion or the location of the insertion in the gene. Only complementary validation by Sanger sequencing could further acquire the information mentioned above, but due to its own constraints, Sanger sequencing is much less sensitive to detect low frequency variations of ITD [7].

Modern advances in next-generation sequencing (NGS) have made it viable to realize FLT3-ITD at single-nucleotide resolution, successfully overcoming the drawbacks of conventional approaches, and minimizing the time and resource waste associated with nonsense mutation detection [8, 9]. However, barriers of NGS in detecting large ITDs and accurately reporting ITD frequencies have been reported. Furthermore, existing methods for identifying insertions and deletions (indels) (e.g., Pindel) can detect small-to medium-sized ITDs, whereas large-sized ITDs (>100 bp) are frequently detected by tools designed to detect structural variations (SVs) [8]. Clearly, tools specifically designed for ITD detection and accurate reporting across the entire size range are lacking.

Hence, in order to develop a reliable tool for accurate clinical assessment of ITD mutations in AML patients of various size ranges and improve prognosis, this study set out to develop a novel NGS-based ITD mutation detection system named “ITDFinder” to achieve rapid detection of small-to large-sized ITDs. The ITDFinder system would also provide comprehensive information including accurate quantification, insertion length, and insertion location.

## Materials and methods

### Sample source

Bone marrow samples from AML patients at the time of initial diagnosis from December 2021 to March 2022 were collected and sent to Shanghai Tissuebank Biotechnology Co., Ltd (China) for high-throughput screening of genes related to hematological disorders for testing the performance of the newly established system. The study was approved by the local ethics committee. All participants signed an informed consent form.

### NGS flow

Each sample was first captured in a certain amount of DNA using a stacked probe, followed by NGS library construction sequencing as previously reported [10]. Then, quality control filtering was performed on each input sample using Fastq (v0.19.5, parameter -c-q30), after which the reads were mapped to the human reference genome hg19 using the Burrows-Wheeler Alignment (BWA) tool v0.7.17, and the output SAM file was compressed, sorted, and indexed through SAMtools v1.10 [11–13]. Finally, software analysis was performed as follows: the obtained BAM file was partially aligned to FLT3 exons 14–15 (1787–2024 in ENST00000241453, 1705–1942 in coding sequence [CDS]) of the soft-clip (SC) reads. Each SC was classified according to its position at the beginning (sSC) or end (eSC) of the alignment region [13–15], and then aligned returned to the target region using local alignment, with the obtained terminal position acting as the anchor of the reads and those scoring <50% discarded. The section enclosed by the alignment position given by BWA and the local alignment was the ITD candidate position determined by means of the reads.

### Dilution method

To analyze the presence of FLT3-ITD mutations, DNA and cDNA samples were subjected to fragment analysis using PCR followed by capillary electrophoresis. Based on the capillary electrophoresis results from patient samples, DNA extracted from the K562 cell line, which is known to lack the FLT3-ITD mutation, was used to dilute the patient samples. This created a series of diluted samples with varying ITD proportions. For the detection of FLT3-ITD mutations, nested PCR was performed on the prepared DNA and cDNA samples. The first round of PCR was followed by a second round of nested PCR, which enhances the sensitivity and specificity of the detection. The PCR products were then analyzed by capillary electrophoresis to determine the presence and size of the ITD mutations. PCR products containing the FLT3-ITD mutations were selected for library preparation. Libraries were constructed according to the manufacturer’s protocol and sequenced using a high-throughput next-generation sequencing platform (e.g., Illumina).

### Establishment of a SC-based short-read tandem duplication recognition system

Considering that SC is a prominent feature for the occurrence of short-read tandem duplication, we first located the position of ITD on the genome starting from SC [16].

### Statistics of SC in alignment files

The BAM file records the alignment results between offline reads and reference genomes. When aligning the reads with the reference genome, if one end of the reads mismatched with the reference genome, it was recorded as a SC in the BAM file. Information on the reads where SCs occurred in the alignment records and their corresponding positions to the reference genome were collected.

### Positioning the beginning and end of ITD

**FIGURE 1:**
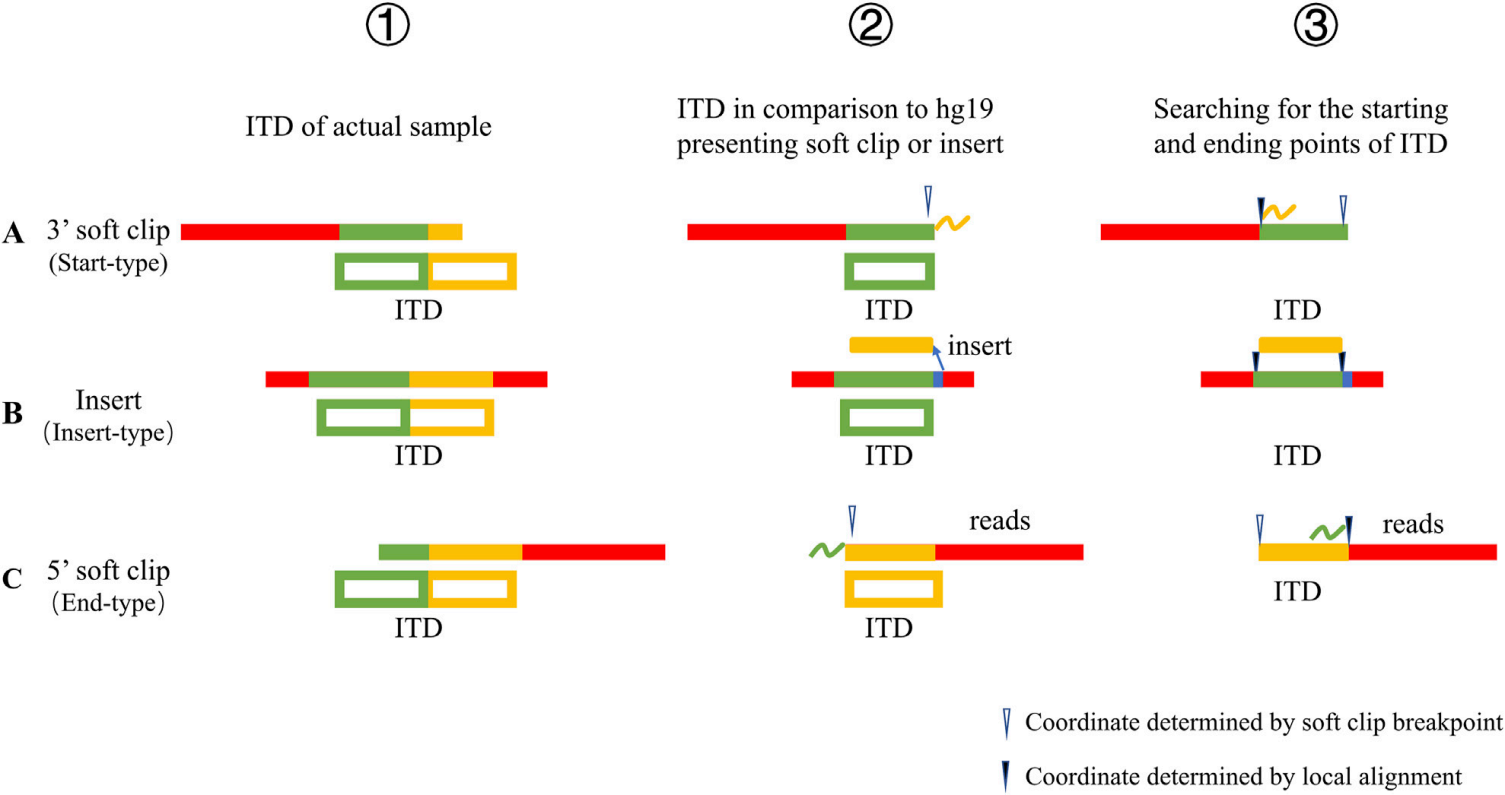
Explanation of the principle of positioning the starting and end points of ITD. **(A)** Start-type: The breakpoint generating the SC suggested an end point of the ITD (②). Since this SC (yellow, ①) corresponds to the front end of the second component of the ITD, and these two components are identical, this SC could certainly be compared to the front end of the first component (③). In this way, the starting point of the ITD could be determined. **(B)** Insert-type: Since the ITD was an additional copy of the multiplied section, its comparison with hg19 was displayed as an insert afterwards. By aligning the inserted section near the insertion point, the starting and end points could be found. **(C)** End-type: The breakpoint generating the SC could suggested a starting point of the ITD (②). Since this SC (green, ①) corresponded to the back end of the first component of the ITD and the two components were identical, this SC could certainly be compared to the back end of the second component (③).

The reads were randomly covered to each position of the ITD, and for the reads falling on the junction of two components of the ITD, a SC could be formed by comparison with hg19; for the reads completely covered to both components of the ITD, an insert could be formed by comparison with hg19. The three specific types of ITDs were composed of Start-type (Figure 1A), Insert-type (Figure 1B), and End-type (Figure 1C). In this way, the end point of the ITD could be determined.

### Determination of the starting and end points of ITDs

In accordance with experimental principles, each reads was known to determine the starting and end points of an ITD, that is, this ITD could be regarded as a candidate ITD, and the corresponding reads could be considered evidence of a candidate ITD. After filtering all candidate ITDs, the number of reads each candidate ITD owned was calculated, and the authenticity of the ITD was judged by the number of reads. If there were more than two different types of reads pointing to the same ITD, the likelihood of the position being a true ITD was high. The matches produced due to chance sequence similarity, often with only one-sided evidence, were recorded as “only start” or “only end” in the result file.

### Initial parameter adjustment settings

After large-scale sample testing, the following initial parameters (thresholds) were chosen to maintain appropriate sensitivity and specificity during alignment: *min_sc_length*, if the SC or insertion was below the value, then the subsequent alignment was excluded; the penalty points for *gap_open* and *gap_extend*, respectively, in the alignment; *min_score_ratio* and *min_sc_aln_ length*, respectively, representing the alignment quality and length filtering threshold. Parameters of the output included: *base_level_num*, which was used to screen for low-support ITDs and then pair different types of evidence-based candidate ITDs; *prominent_level*, defined as high confidence positive if above this threshold; and *uncertain_threshold*, a candidate ITD supported only by unilateral evidence that was not lower than this threshold was classified as such, otherwise it was classified as negative.

### Output results

**FIGURE 2:**
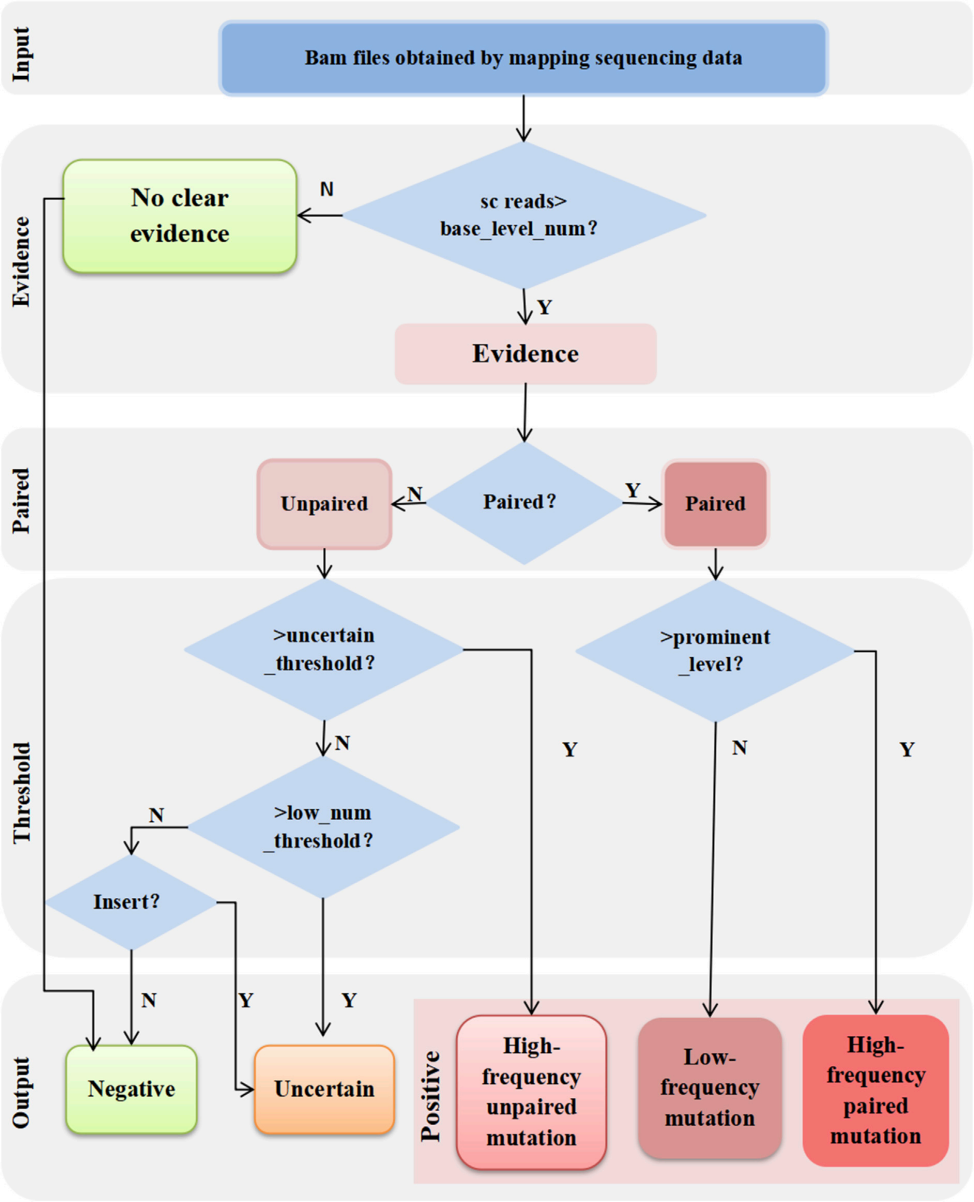
Output results logic diagram. Candidate ITDs with the number of supported reads below the threshold of *base_level_num* are filtered out. Those above the threshold of *base_level_num* are paired with supporting evidence to see which candidate ITDs are supported by multiple types of evidence. Candidate ITDs that are supported by multiple types of evidence are output as positive, while those that cannot be paired are considered as ITDs supported by only unilateral evidence. For ITDs supported by unilateral evidence only, if it meets the threshold of *uncertain_threshold*, the output is positive or indeterminate according to whether it meets the threshold of *prominent_level* for capillary electrophoresis verification; those below the threshold of *uncertain_threshold* are judged as indeterminate if they are insert type, otherwise they are judged as negative.

The results satisfying the set parameters (thresholds) were output according to the logic, as shown in Figure 2.

### Performance validation

The performance validation of the detection method was conducted by evaluating its sensitivity and accuracy in identifying tandem duplications within a simulated dataset. We established the lower detection limit by modifying the reference values to align with the performance of capillary electrophoresis, setting a sensitivity threshold between 3% and 5% (median 4%) based upon previous studies. [17]. Data simulation was conducted via the dwgsim program (version 0.1.11). The CDS of the FLT3 gene (ENST00000241453) served as the wild-type reference sequence. The CDS encompasses nucleotide locations 1705–1942, and a tandem duplication was modelled by introducing a piece of variable length (5-70 base pairs) at a fixed location inside the FLT3 CDS (position 1610) of the hg19 reference genome. This facilitated the creation of simulated short-read tandem duplication mutations of varying durations. The simulation was regulated with the -c parameter in dwgsim, which specifies the sequencing depth. A coverage range of 900X to 1300X was applied to the simulated data, and the impact of varied mutation rates was analyzed by combining variable fractions of wild-type and mutant reads. The simulation outcomes, displayed as combinations of wild-type and mutant reads, facilitated the evaluation of detection capabilities at various mutation frequencies and sequencing depths. This method validated the system’s capacity to identify tandem duplications with excellent sensitivity, even at reduced mutation rates.

### Validation of the applicability to other pathogenic tandem duplication mutations

To evaluate the system’s efficacy regarding additional harmful tandem duplication mutations beyond FLT3-ITD, we concentrated on haematologic malignancies and a range of tumour types. Utilising previously validated BCOR-ITD sequences for clear cell sarcoma of the kidney (CCSK), we produced simulated data for four sequence types employing dwgsim v0.1.11 software [18]. We investigated histone-lysine N-methyltransferase 2A (KMT2A)-partial tandem duplication (PTD) as a prospective therapeutic target and biomarker for minimum residual disease in acute myeloid leukaemia (AML) and myelodysplastic syndrome (MDS). [19]. A recent investigation yielded 25 clinically validated KMT2A-PTD sequences, from which four were randomly selected for the creation of simulation data. We established varying sequencing depths for each sequence class, specifying mutation rates for each gradient. Each simulation was conducted five times to verify reliability. This methodology illustrates the system’s wider application to other pathogenic tandem duplications, hence augmenting its utility in clinical contexts.

### Statistical analysis

Count data were described using absolute frequencies and percentages. Bland-Altman plots, scatter plots and mosaics were generated to analyze the methodological agreement of this new system with capillary electrophoresis. All statistical analyses were performed with GraphPad Prism 8 and R (4.2.0) software.

## Results

### Presentation form of the new system

**FIGURE 3:**
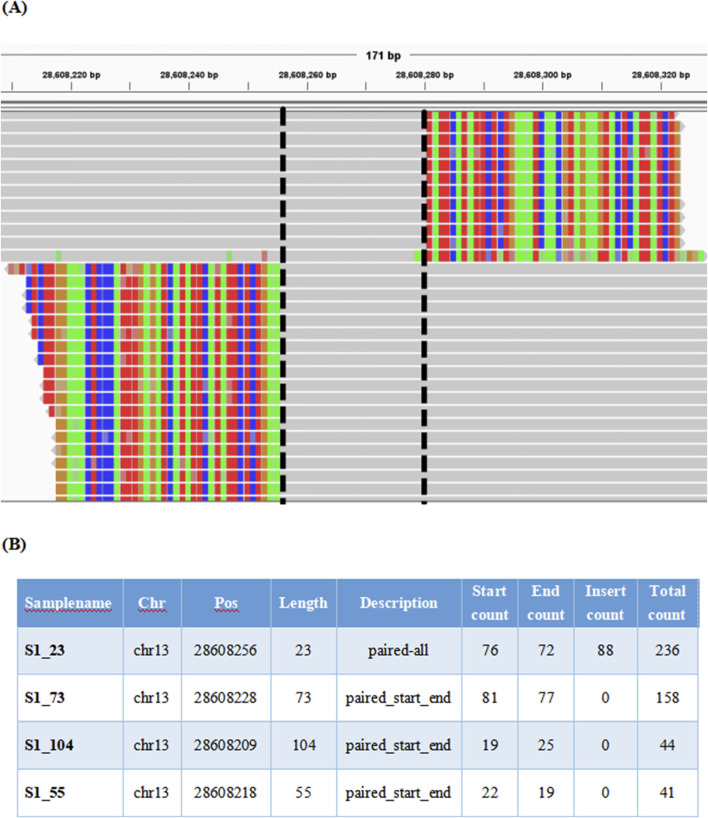
Example of redundant FLT3-ITD lists generated by the new system. **(A)** Integrative Genomics Viewer screenshot of a positive sample (FLT3-ITD of 20 bp at chr13: 28,608,256–28,608,276). Reads covering ITD are marked as colored SC. The colored strips on either side of the dashed line represent the SC segments, and the part between the two dashed lines represents the short-read tandem duplication region. The black dotted line represents the left and right breakpoints formed by aligning these reads to the reference genome, and the sequences between the two breakpoints represent the duplicated fragments. **(B)** FLT3-ITD lists detected by the new system. Terms in the header are explained as follows: *chr* represents the chromosome name; *pos* represents absolute chromosome position; *length ITD* represents the length of ITD; *description* represents the type of reads supporting this ITD; *start count* represents the count of Start-type reads; *end count* represents the count of End-type reads; *insert count* represents the count of Insert-type reads; and *total count* represents the total number of reads supporting this ITD.

We developed an innovative NGS-based system named “ITDFinder” for accurate detection of pathogenic tandem duplication mutations. The presentation of tandem duplication region reads in positive samples in Integrative Genomics Viewer is shown in Figure 3A. In addition, a FLT3-ITD had multiple mutation items with consecutive insertion positions. An example of the output file for positive sample results reported by the ITDFinder system was displayed in Figure 3.

### Performance evaluation of the new system

#### Lower detection limit

When the mutation rate of the test samples was below 4%, some of the samples were not detected (marked in red), whilst the mutation rate above 4% could be detected, thus the lower detection limit of the system was 4%, corresponding to a sequencing depth of 1000X (Table 1).

**TABLE 1:** Validation results of the lower detection limit of this new system.

ITD length	Simulated sequencing depth (X)	Mutation rate (%)					
		2	4	6	8	10	20
5	900	19	37	34	71	99	163
	1100	26	45	61	76	80	209
	1300	24	55	66	96	113	220
10	900	15	31	43	66	53	150
	1100	13	29	49	83	78	203
	1300	25	44	66	80	103	249
20	900	Below threshold	22	45	65	68	167
	1100	16	35	34	75	100	213
	1300	17	42	63	91	123	246
30	900	Below threshold	27	46	65	57	164
	1100	15	37	59	94	96	209
	1300	Below threshold	42	71	86	119	252
40	900	Below threshold	14	38	65	92	172
	1100	Below threshold	33	59	72	100	208
	1300	Below threshold	39	63	92	121	225
50	900	22	33	31	57	89	165
	1100	11	42	56	71	87	218
	1300	13	48	70	107	120	222
60	900	12	34	43	69	88	155
	1100	Below threshold	29	64	83	99	215
	1300	12	49	75	98	121	213
70	900	Below threshold	30	52	56	81	195
	1100	17	38	58	81	105	204
	1300	13	49	82	87	124	237

### Methodological consistency comparison

**FIGURE 4:**
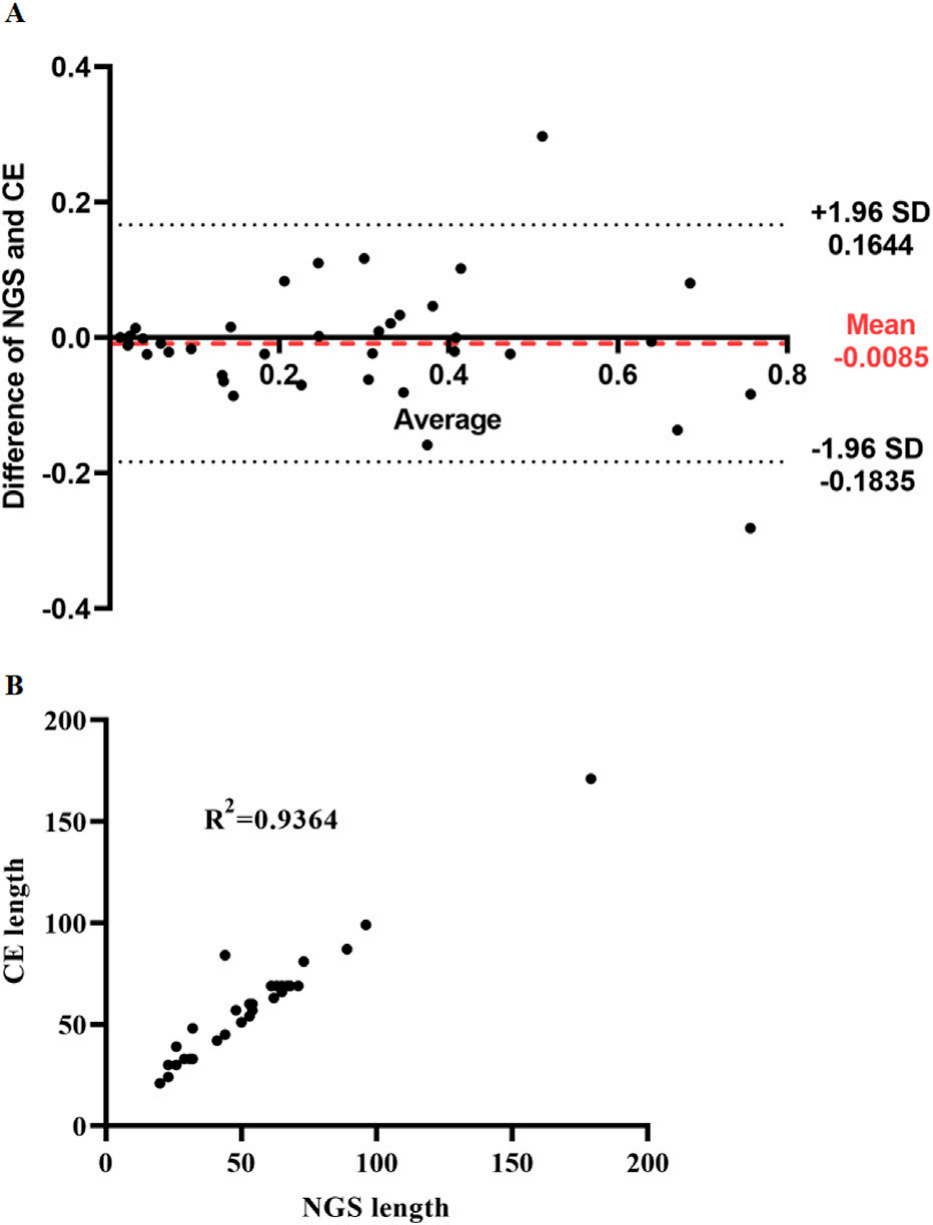
Comparison of the methodological consistency of this new system with capillary electrophoresis. **(A)** The difference of NGS and CE. **(B)** CE length.

The mean difference between the mutation rate detection results of the ITDFinder system and capillary electrophoresis was −0.0085 (range, −0.1835 to 0.1644), indicating that ITDFinder was in good consistency with capillary electrophoresis and feasible for ITD quantification (Figure 4A). In addition, there was a good correlation between the length of ITDFinder and capillary electrophoresis, indicating that ITDFinder has the ability to detect multiple sizes of ITDs (Figure 4B).

### Clinical case validation

Among the 1,032 clinical samples used for validation, 51 samples (4.94%) were completely consistent with the positive capillary electrophoresis validation by the ITDFinder system, all of which were positive. Of the remaining samples with negative capillary electrophoresis verification results (n = 981), 96.3% were recognized as negative by ITDFinder, with the two methods in agreement; 3.4% were identified as positive by ITDFinder, contradicting the capillary electrophoresis verification results; and the remaining 0.3% were inconclusive (Table 2). Thus, the overall agreement rate between the two approaches used for characterization was 96.5%. It was assumed that the both ways are equivalent in most cases of determination, but the ITDFinder system can additionally identify positive mutation cases that cannot be measured by means of capillary electrophoresis.

**TABLE 2:** Comparison of the qualitative results of this new system with capillary electrophoresis for clinical samples.

		Capillary electrophoresis (CE)				Total
		Positive		Negative		
		N	%	N	%	
New system	Positive	51	100%	33 (FP)	3.4% (FPR)	84
	Negative	-	-	945	96.3%	945
	Uncertain	-	-	3	0.3%	3
Total		51	100%	981	100%	1032

FP, false positive; FPR, false positive rate.

The above results also indicated that ITDFinder was expected to save 96.3% of the workload (i.e., its determination results were used directly without capillary electrophoresis verification), while the remaining 3.7% of the samples were categorized as negative for capillary electrophoresis verification (meaningless retest).

### Validation of the applicability of the new system to BCOR-ITD and KMT2A-PTD

**FIGURE 5:**
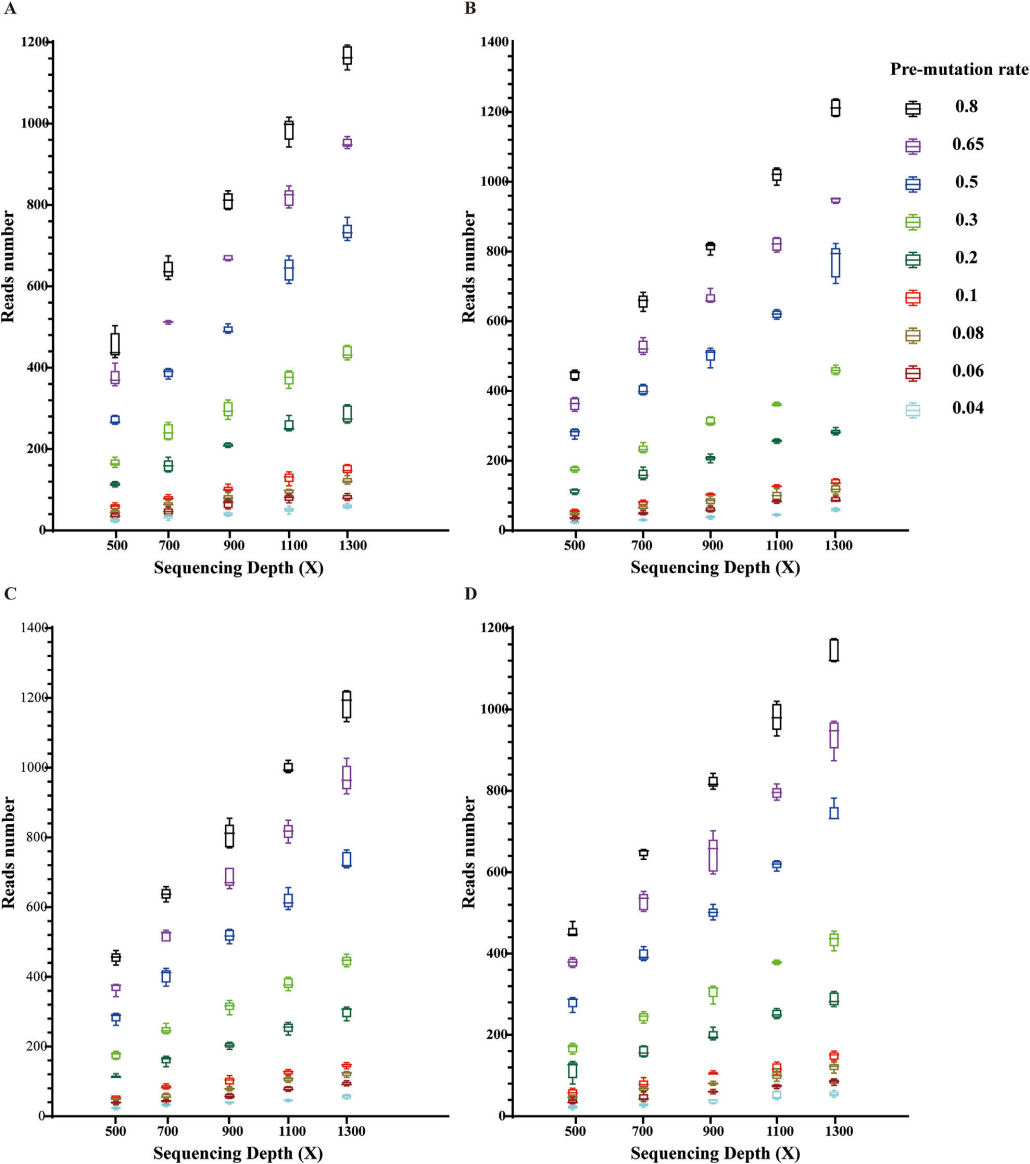
Validation of the applicability of this new system in BCOR-ITD mutation detection. Each box indicates each repetition; different colors indicate different preset mutation frequencies in the range of 0.04–0.8. **(A)** Reptition 1, **(B)** Reptition 2, **(C)** Reptition 3, **(D)** Reptition 4.

**FIGURE 6:**
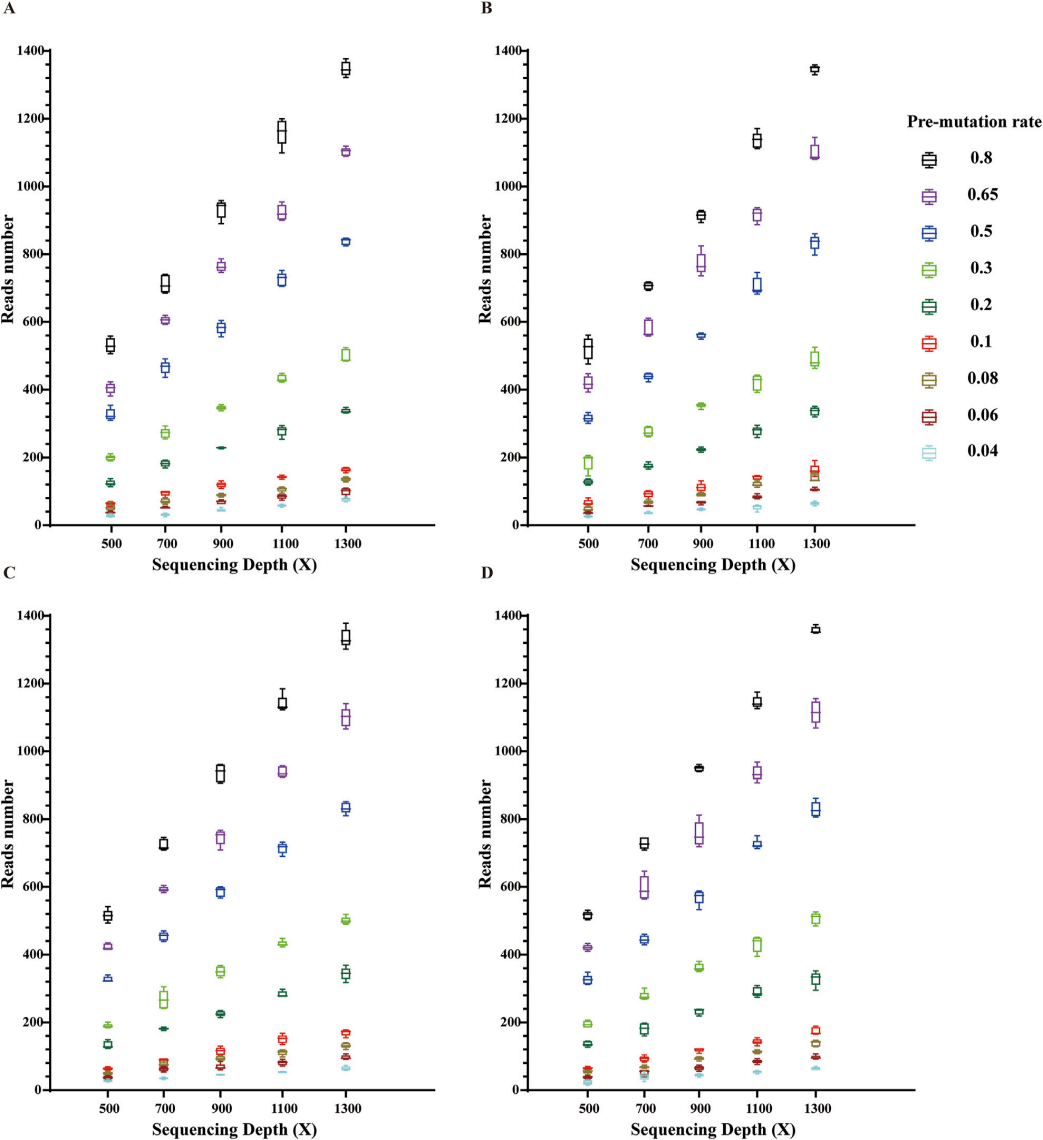
Validation of the applicability of this new system in KMT2A-PTD mutation detection. Each box indicates each repetition; different colors indicate different preset mutation frequencies in the range of 0.04–0.8. **(A)** Reptition 1, **(B)** Reptition 2, **(C)** Reptition 3, **(D)** Reptition 4.

Using clinically validated typical ITD mutations in hematologic and non-hematologic diseases that have been mentioned as simulation objects, the above ITD sequences were made into simulation data and used to verify the applicability of the ITDFinder system to ITDs other than FLT3-ITD. Based on the data simulation results, ITDFinder can observe BCOR-ITD (Figure 5) and KMT2A-PTD mutations (Figure 6) with mutation depths in the vary of 500–1300X and mutation rates in the range of 0.04–0.8. Together with the aforementioned results, they collectively demonstrated that ITDFinder is a reliable tool specifically for ITD detection, not only for full-sized ITD mutations including FLT3-ITD, but also for mutations with longer sequences (e.g., PTD).

## Discussion

In the present study, we developed an innovative NGS-based pathogenic tandem duplication mutation precision detection system, called as “ITDFinder”, beyond capillary electrophoresis and towards multi-sized mutation length. Since AML with FLT3-ITD mutations yields a high prevalence, rapid relapse rate, and generally poor prognosis, their early identification does have a considerable potential to ameliorate the aforementioned events [20–23]. Furthermore, ITD is the most common type of FLT3 mutation in AML patients, raising the bar for understanding FLT3-ITD and, by extension, AML pathogenesis [24].

Given the nature of heterogeneity, routine AML screening relies on a variety of technical equipment at the cytogenetic and molecular levels. NGS technology, amongst others, obtains and validates results comparable to many conventional molecular and cytogenetic analyses by means of inspecting the giant quantity of genomic information obtained in a single assay with extensive use of multiple bioinformatics algorithms [25]. Compared with regular capillary electrophoresis, NGS allows the identification of single nucleotide and gene mutations with lower detection limits and presents more objective FLT3-ITD allele load quantification, with the benefits of short running time, low fee of large-scale pattern detection, and flexible library preparation and analysis strategies to tackle the challenges of difficult-to-sequence genomic fractions [26, 27]. In this study, FLT3-ITD was detected based on NGS, which could assist apprehend the genetic mutation composition of AML, in turn guide the classification of AML by mutation, and is expected to more accurately combine FLT3-ITD with adverse prognosis in AML patients [5].

The sequence and length of ITD mutations are heterogeneous and vary by patient. Research has shown that longer ITD in patients with positive FLT3-ITD mutations is associated with shorter overall survival and relapse-free survival [28]. Current study found a poor correlation between risk and high mutation load in the FLT3-ITD mutation subgroup [29]. In contrast, the existing detection tools suffer from poor accuracy, inapplicable to low-frequency variants, and unable to notice larger ITD frequencies. FLT3-ITD detection by NGS is challenging primarily because standard bioinformatics algorithms are not optimized for large insertion/deletion (>20 bp) detection. Upon optimization and validation, the NGS system was found to be 100% consistent in detecting FLT3-ITD in presence of variable size (3–231 bp) and insertion sites [6]. Due to the dependence of small- and large-sized ITDs on the detection of insertion and structural mutations, neither of them could be achieved with currently available software [5]. Hence, in order to settle these issues, ITDFinder for accurate detection of FLT3-ITD mutations was created, based on the NGS data collected from existing AML samples, which can rapidly detect full-size ITD mutations, and negative results can be directly used as a reference after comparison with capillary electrophoresis. According to reports, an increase in the frequency of FLT3-ITD mutations in refractory AML predicted a decrease in complete remission rate and overall survival rate after relapse [30]. Therefore, precise detection of ITD mutations using NGS may provide a basis for studying the molecular mechanisms of refractory or relapsed leukemia, and open up a new perspective for dynamic risk assessment of AML. Notably, both FLT3-ITD and KMT2A-PTD in AML patients involve the adverse outcome-related molecular features [31]. Recent evidence emphasizes that considering KMT2A-PTD mutations as a potential adverse prognostic factor for AML patients [32]. Therefore, we confirmed the performance of the ITDFinder system for the detection of other types of ITD (i.e., BCOR-ITD) and PTD (i.e., KMT2A-PTD) through simulated data.

Importantly, by comparing eight available and most representative software platforms for detecting FLT3-ITD (Table 3) [9, 33–40], it is not difficult to see that the shortcomings of the above tools can be addressed through our tool ITDFinder. Specifically, ITDFinder has the ability to accurately determine the percentage of tandem repeat mutations in multiple diseases, not limited to AML. In addition, it can quickly identify both large and small ITD mutations; its short runtime allows it to identify full-sized ITD mutations, such as FLT3-ITD.

**TABLE 3:** Representative software programs for detecting FLT3-ITD.

Tool	Theory	Strength	Limitation
Pindel [33]	Split-read strategy using a pattern growth algorithm	- Ability to detect breakpoints of large deletions and medium-sized insertions from paired-end short reads - Ability to detect short (10–200 bp) tandem duplications and structural variants	- Low frequency of detected mutations
ITDetector [34]	Assembly strategy	- effective avoidance of deviations caused by alignment errors	- Inability to detect ITDs with more than two duplicates - Only for somatic ITD detection, not for germline detection
ITDseek [35]	Soft-clipping with the alignment information	- Reporting all insertions of FLT3 exons 14 and 15 in the result as itds - Dependent on the -M parameter of BWA-MEM to mark shorter split hits as secondary	- Lack of research or evaluation on unreported hybridization capture sequencing data - Inability to differentiate shorter insertions and tandem repeats
getITD [36]	High-quality sequencing reads aligning to the reference genome to identify insertions	- Detecting and fully annotating all tested itds - Achieving 100% sensitivity and specificity	- Inability to parse large files, requiring manual extraction of reads in a certain region - The upper limit of detected ITD length affected by sequencing data length
ScanITD [37]	Soft-clipping with the alignment information	- The string rotation method, distinguishing between insertions of new sequences and duplications of genome sequences	- Inability to detect complex types of FLT3-ITD
FLT3_ITD_ext [38]	Central clustering-based greedy algorithm SUMACLUST	- Ability to effectively solve the shortcomings of existing methods that underestimate mutation frequency	- Inability to identify large, purely non-templated insertions
ABRA2 [39]	Assembly strategy	- Good performance on short Indels and FLT3 ITDs with <100 bases	- Significantly delay in result generation, owing to the increased processing time caused by compute resource
FiLT3r [9]	k-mers	- Ability to detect duplications in any gene once the reference sequence is known - More precise (neither false-positive nor false-negative)	- Inability to detect duplications that would occur at the beginning or end of a read

Overall, ITDFinder has two significant advantages. First, negative results achieved with ITDFinder are roughly equivalent to negative capillary electrophoresis results, thereby removing the necessity for capillary electrophoresis experiments (almost 97% of samples based on high volume validation) and shortening NGS cycle times. Additionally, as a system specifically designed for ITD detection, it is additionally appropriate for accurate detection of tandem duplication mutation ratios in different diseases, which includes BCOR-ITD and KMT2A-PTD. The former is essential for the diagnosis and therapeutic strategy of CCSK [18]; and the latter is valuable as an AML causative gene in the dynamic monitoring of tumor burden and can be used as one of the markers of disease onset, progression and clonal evolution [37, 41].

The limitation of the ITDFinder system is that it cannot fundamentally improve the limitation of the NGS technology’s filtering operation, which may change the ratio of normal reads to wild-type reads, often leading to uncontrollable errors in the results, ambiguous judgments on the normal or mutant type of reads [42], leaving its ability to calculate accurately to be improved. Further and substantial advancements in this field may be achieved in the future by attempting to utilise approaches such as triple sequencing [42].

## Conclusion

The ITDFinder system for accurate detection of pathogenic tandem duplication mutations is equivalent to capillary electrophoresis assays in most cases of determination and can additionally identify positive mutation cases that cannot be measured by the assay, saving 96.3% of the workload. ITDFinder is capable of detecting not only full-size ITD mutations including FLT3-ITD, but also PTD mutations, and offers significant potential for accurate clinical assessment of ITD mutations in AML patients, predicting prognostic risk, and optimizing therapy options.

## Data Availability

The original contributions presented in the study are included in the article/supplementary material, further inquiries can be directed to the corresponding author.
